# Novel bioactive glass based injectable bone cement with improved osteoinductivity and its *in vivo* evaluation

**DOI:** 10.1038/s41598-017-03207-9

**Published:** 2017-06-15

**Authors:** Tengjiao zhu, Huihui Ren, Ailing Li, Bingchuan Liu, Caiyun cui, Yanmei Dong, Yun Tian, Dong Qiu

**Affiliations:** 1 Orthopedic Department, Peking University Third Hospital, Beijing, 100191 P.R. China; 2 Orthopedic Department, Peking University International Hospital, Beijing, 102206 P.R. China; 3 Beijing National Laboratory for Molecular Sciences, State Key Laboratory of Polymer Physics and Chemistry, Institute of Chemistry, Chinese Academy of Sciences, Beijing, 100190 P.R. China; 4 University of Chinese Academy of Sciences, Beijing, 100190 P.R. China; 5 Department of Cariology and Endodontology, Peking University School and Hospital of Stomatology, Beijing, 100081 P.R. China

## Abstract

Recently, more and more attention has been paid to the development of a new generation of injectable bone cements that are bioactive, biodegradable and are able to have appropriate mechanical properties for treatment of vertebral compression fractures (VCFs). In this study, a novel PSC/CS composite cement with high content of PSC (a phytic acid-derived bioactive glass) was prepared and evaluated in both vitro and vivo. The PSC/CS cement showed excellent injectability, good resistance to disintegration, radiopacity and suitable mechanical properties. The *in vitro* test showed that the cement was bioactive, biocompatible and could maintain its shape sustainably, which made it possible to provide a long-term mechanical support for bone regeneration. Radiography, microcomputed tomography and histology of critical sized rabbit femoral condyle defects implanted with the cements proved the resorption and osteoinductivity of the cement. Compared with the PMMA and CSPC, there were more osteocyte and trabeculae at the Bone-Cement interface in the group PSC/CS cement. The volume of the residual bone cement suggested that PSC/CS had certain ability of degradation and the resorption rate was much lower than that of the CSPC cement. Together, the results indicated that the cement was a promising bone cement to treat the VCFs.

## Introduction

Vertebral compression fracture (VCF) is probably the most common complication in patients with osteoporosis, with an estimated 1.4 million new fractures occurring every year worldwide^1^. The minimally invasive surgeries of percutaneous kyphoplasty (PKP) and percutaneous vertebroplasty (PVP) are safe and effective for VCFs. Both procedures involve percutaneous injection of the setting dough of an injectable bone cement either directly to the fractured vertebral body (PVP) or to a void created in it by an inflatable bone tamp (PKP). Thus, injectable bone cement is essential in both procedures. Currently available injectable bone cements in clinic mainly include poly(methyl methacrylate) (PMMA), calcium sulfate cement (CSC) and calcium phosphate cement (CPC)^2^.

PMMA is the most widely used injectable cement in PVP and PKP, because of its suitable curing behavior and easiness of handling. Although there have been great success, several drawbacks still need to be further improved. PMMA is not bio-resorbable, therefore cannot be replaced by new bone but forms an implant-host interface. PMMA also lacks active bonding with surrounding bone, thus the long-term mechanical stability of implant-host interface is still unsatisfactory^3^. Besides, the intensive exothermic effect in the polymerization process may lead to thermal necrosis in surrounding tissues^4^. Moreover, differences in mechanical strength between PMMA and the adjacent vertebral body are often found to cause adjacent vertebral fractures^5^.

CSC, with self-setting ability, has enjoyed a long history of clinical use as injectable bone augmentation^6, 7^. It is virtually complete resorbable *in vivo*, thus can overcome the long-term stability issue often faced by PMMA cement. These properties make it possible to serve as a delivery vehicle for drugs, growth factors or as a soluble additive to modulate the porosity or biodegradable rate when blended with other biomaterials^6, 8^. Despite these virtues, CSC has been criticized for its rapid resorption rate. Studies have shown that the degradation of CSC implant is much faster than bone ingrowth, which makes it fail to provide adequate mechanical support^3, 6, 9^.

Calcium phosphate has excellent biocompatibility, bone conductivity, and slower degradation rate than calcium sulfate. When mixed with calcium sulfate, injectable bone cements can still be formulated, which degrade slower and stimulate bone growth, thus are expected to solve the long-term mechanical support issue^8^. This strategy has obtained certain success and clinical products have been developed based on these combinations, for example Genex®, a widely used resorbable bone cement for bone defect filling. However, the resorption rate is still too fast and further collapse after treatment of vertebral compression fractures often takes place, thus the calcium phosphate-calcium sulfate combination only finds very limited success in PKP or PVP procedures^10, 11^. Further development should focus on slowing down degradation and improve osteogenic property of bone cement, in order to maintain a sufficient mechanical support until the fractured vertebrae recovers.

Bioactive glass (BG) represents one of the most promising artificial bone repair materials, due to their excellent biocompatibility, bioactivity and osteoinductivity^12, 13^. The ions released by BG, especially soluble silicon and calcium ions, were proved to stimulate osteoprogenitor cells at the genetic level and thus promote bone regeneration^12^. Up to date, BG has been successfully used in bone regeneration and dental applications^13, 14^. BG also degrades much slower than calcium phosphate^15^. Therefore, it is imaginable that when BG is blended with calcium sulfate, injectable bone cements with slower resorption and better osteogenic performance could be developed. Indeed, several studies have demonstrated that BG-contained calcium sulfate cements had better osteoinductivity and simulation of bone ingrowth^9, 16, 17^. However, because the conventional BG (i.e. 45S5) increases pH when contacting body fluid, which intervenes with the hydration reactions of calcium sulfate, the content of BG was often very low, i.e. only a few percent by weight. Consequently, it only slightly improved the biogenic property, while had little improvement or sometimes even deterioration on the long term mechanical support, because the curing of calcium sulfate became incomplete^9, 16^.

In this study, we aim to develop an injectable BG/calcium sulfate composite cement with high content of BG by using a novel phytic acid-derived bioactive glass. This bioactive glass with the composition of 10.8%P_2_O_5_-54.2%SiO_2_-35%CaO (mol. %; hereinafter referred to as PSC) can maintain a stable pH when reacting with physiological solution. The hypothesis was that, with higher content of BG, the PSC/calcium sulfate composite cement could show better osteogenic performance and have a slower biodegradation rate to match with the growth of new bone tissue. The physicochemical properties of the cement, such as setting time, injectability, disintegration resistance and mechanical properties were investigated. *In vitro* bioactivity, degradation behavior in simulated body fluid (SBF) and cytocompatibility were studied to evaluate its potential bone integration ability. Eventually, its’ *in vivo* performances were evaluated in a rabbit femoral condyle defect model.

## Results

### Characterization of the cements

**Figure 1:**
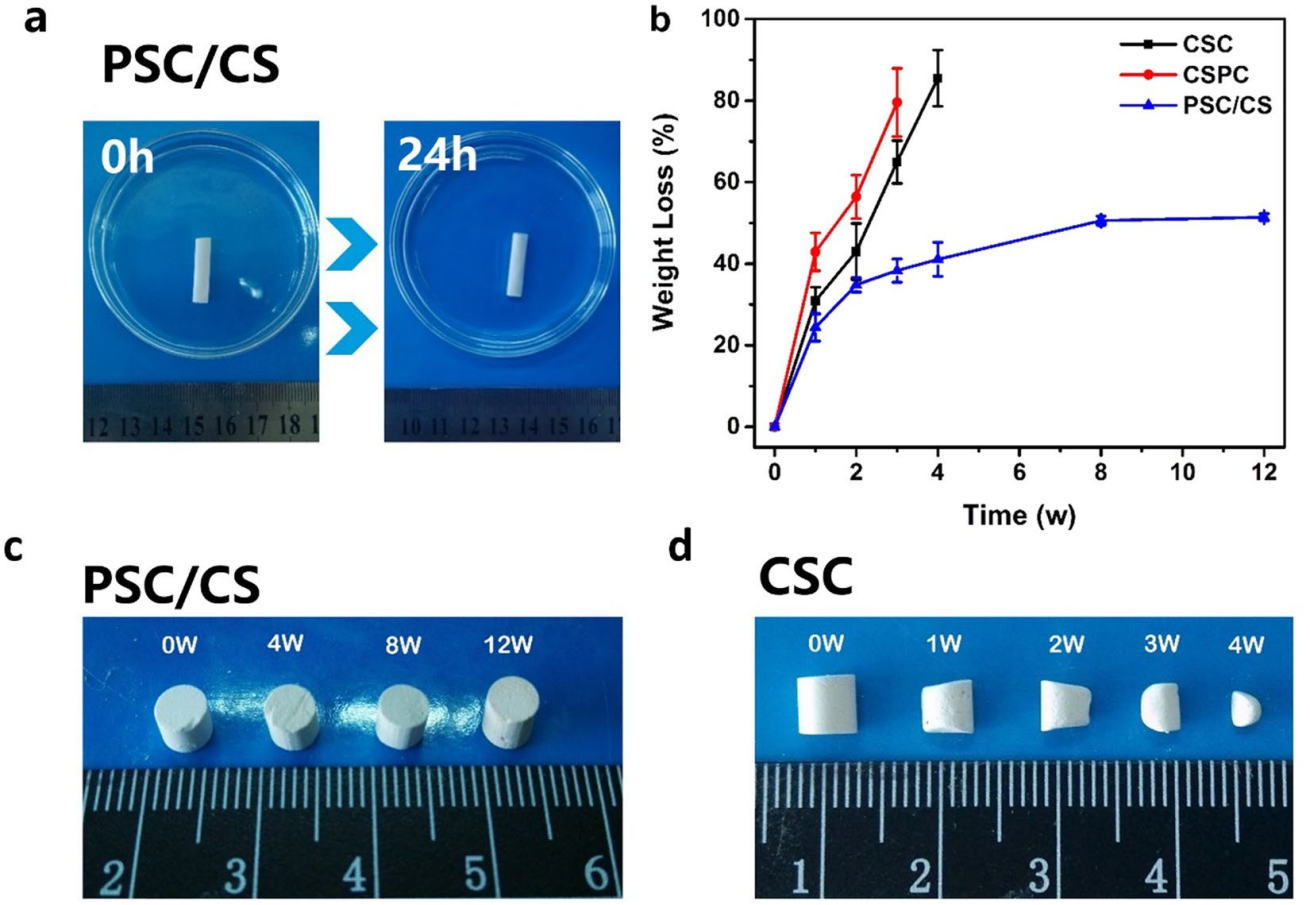
(**a**) The optical image of PSC/CS cement just injected and immersed in PBS for 24 h; (**b**) *in vitro* weight loss profiles of hardened CSC, CSPC and PSC/CS cements after immersion in SBF; the appearance of hardened (**c**) CSC and (**d**) PSC/CS cements after immersion in SBF for different time.

In the present study, a novel bioactive glass based injectable bone cement was developed. As mentioned earlier, the more content of BG, the higher bioactivity of the composite cement. In our preliminary experiments, it was found that when the content of PSC was above 55 wt.%, PSC/CS cement was difficult to set and the compressive strength of resultant material (<2 MPa) was lower than the requirements (2–12 MPa)^18^. Therefore, the PSC content was set to be 55 wt% in the PSC/CS cement through this study. Some physiochemical properties of PSC/CS cement are summarized in Table 1. The injectability of the PSC/CS cement remained above 90% during the first 6 minutes (Fig. S1, Supporting information) and no phase separation were observed when the cements were extruded from the syringe. Therefore, it is possible for them to be used for minimally invasive surgery. From Fig. 1a, it can be seen that the cement could almost remain its initial shape and no obvious decay was observed after immersed in PBS for 24 h. The disintegration resistance of the cements, as determined by Eq. (2), were ~94% (Table 1), which indicated a good resistance to disintegration in PBS. Some weight loss may be caused by the ion release when immersed in PBS (Fig. S2, Supporting information). The compressive strengths (S_c_) and Young’s modulus (E_c_) of the hardened PSC/CS cement were ~2.9 MPa and ~340 MPa, respectively, which were comparable to human trabecular bone (S_c_:2–12 MPa; E_c_:100–500 MPa)^18, 19^.

**Table 1:** Physiochemical properties of PSC/CS cement.

• Injectability	93% ± 2%	• Disintegration resistance	94% ± 1%
• Initial setting time/min.	25 ± 3	• Final setting time/min.	41 ± 2
• Compressive strengths/MPa	2.9 ± 0.3	• Young’s modulus/MPa	340 ± 80

Figure 1b gives the *in vitro* degradation profile of the CSC, CSPC and PSC/CS cements after immersing in SBF. It is seen that the CSC degraded rapidly in SBF (Fig. 1d). The Mass loss of CSC was ~85.5% and the diameter reduced significantly after immersed in SBF for 4 weeks. The degradation rate of CSPC in SBF was even more rapid than CSC, probably due to the interruption of calcium sulfate hardening by calcium phosphate. It is generally accepted that a cement with a degradation rate faster than that of bone ingrowth, such as CSC, is not suitable because it does not provide long-term mechanical support and the space left after its degradation will restrict the growth of newly formed bone^6, 20^.

**Figure 2:**
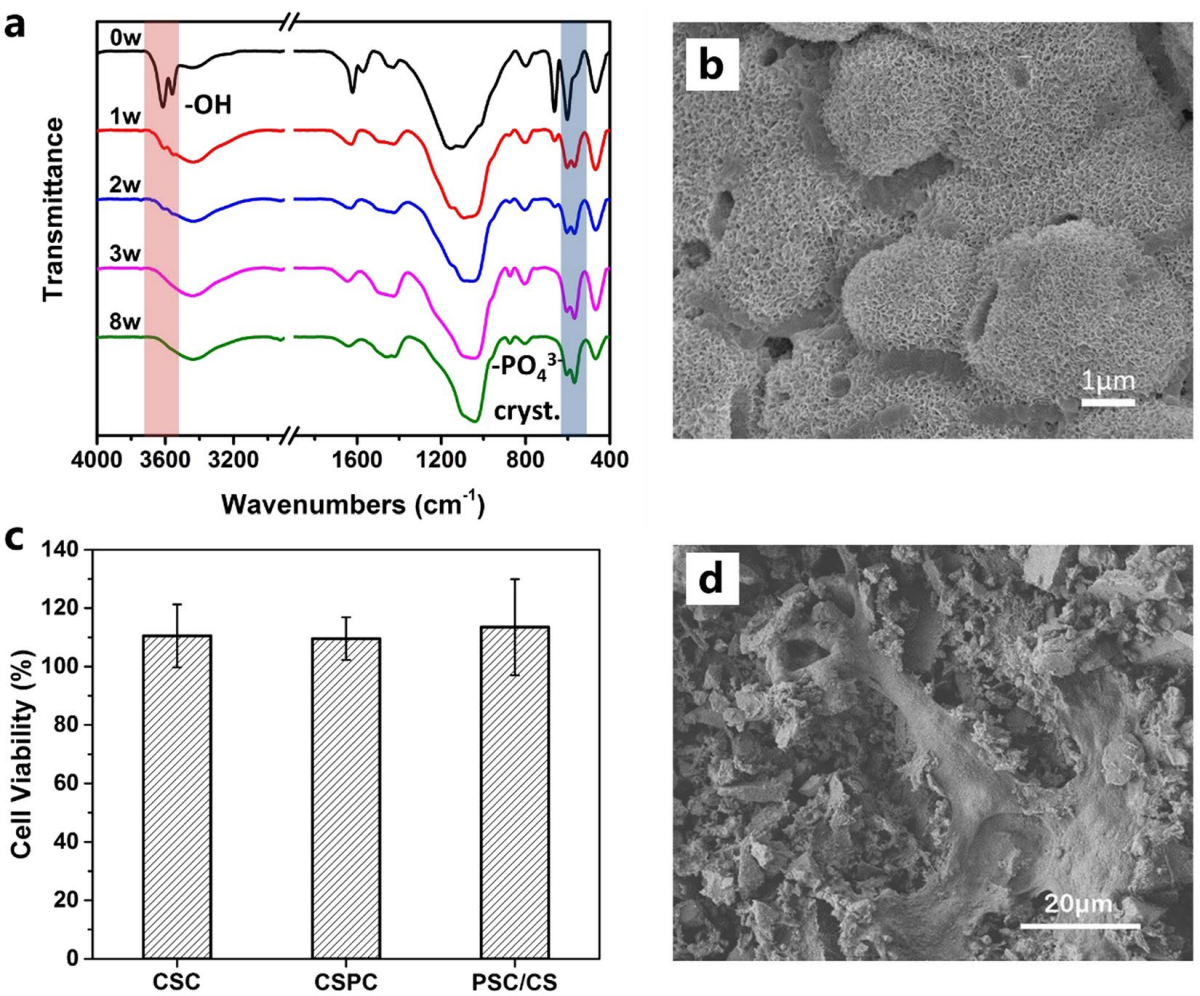
(**a**) FTIR spectra of the PSC/CS cement after immersion in SBF for 0, 1, 2, 3 and 8 weeks. (**b**) SEM images of PSC/CS cement after immersion in SBF for 8 weeks. (**c**) MTT assay for proliferation of MG63 cells after culturing for 48 h. (**d**) The MG63 cell morphologies on PSC/CS cement after culturing for 24 h.

While for PSC/CS cement, its weight loss increased rapidly within the first 3 weeks, after which it only increases with a much slower rate and finally reaches a plateaued at ~52% weight loss (at about 7 ~ 8 weeks). Interestingly, the diameter of PSC/CS specimen had virtually no change and its shape remained almost the same in the whole experimental period (Fig. 1c), which might be caused by the formation of interconnected network of formed hydroxyapatite (HA) upon the reaction of PSC with SBF. Its weight loss was the comprehensive effect of calcium sulfate degradation, PSC degradation and HA formation. The disappearance of calcium sulfate peaks and the appearance of HA peaks on the FTIR spectra provide experimental evidence for the above conjecture (Fig. 2a).

### *In Vitro* Bioactivity and Cell Biocompatibility

After incubation in SBF, the peaks at 607 cm^−1^ and 567 cm^−1^ appeared on the FTIR spectra (Fig. 2a), which could be the evidence of crystalline phosphate, confirming the formation of HA. Figure 2b shows that dense hemispherical and needle like HA crystals was formed on the cement surface. The hemispherical and needle like HA crystals were consistent with previous reports^21^ and in agreement with the results of FTIR. It has been generally accepted that the bone-bonding ability of a material can be evaluated by examining the HA formation on its surface in SBF. Therefore, the PSC/CS cements are potentially bioactive.

The MG63 cell morphologies on the cement surfaces are illustrated in Fig. 2d. After culturing for 24 h, MG63 cells showed a spindle-like or polygonal morphology and spread well on the surfaces of PSC/CS cement, suggesting MG63 cells were able to grow and proliferate well on the samples. The MTT assay was adopted to quantitatively assesse the cell viability. Compared to the control groups (the blank groups, CSC and CSPC), the cell proliferations of PSC/CS cement were slightly higher and no significant difference was observed (p > 0.05). These results demonstrated the PSC/CS cement showed good cell compatibility and had no cytotoxic effect on MG63 cells.

### Bone regeneration and resorption of cement *in vivo*

#### X-ray analysis

**Figure 3:**
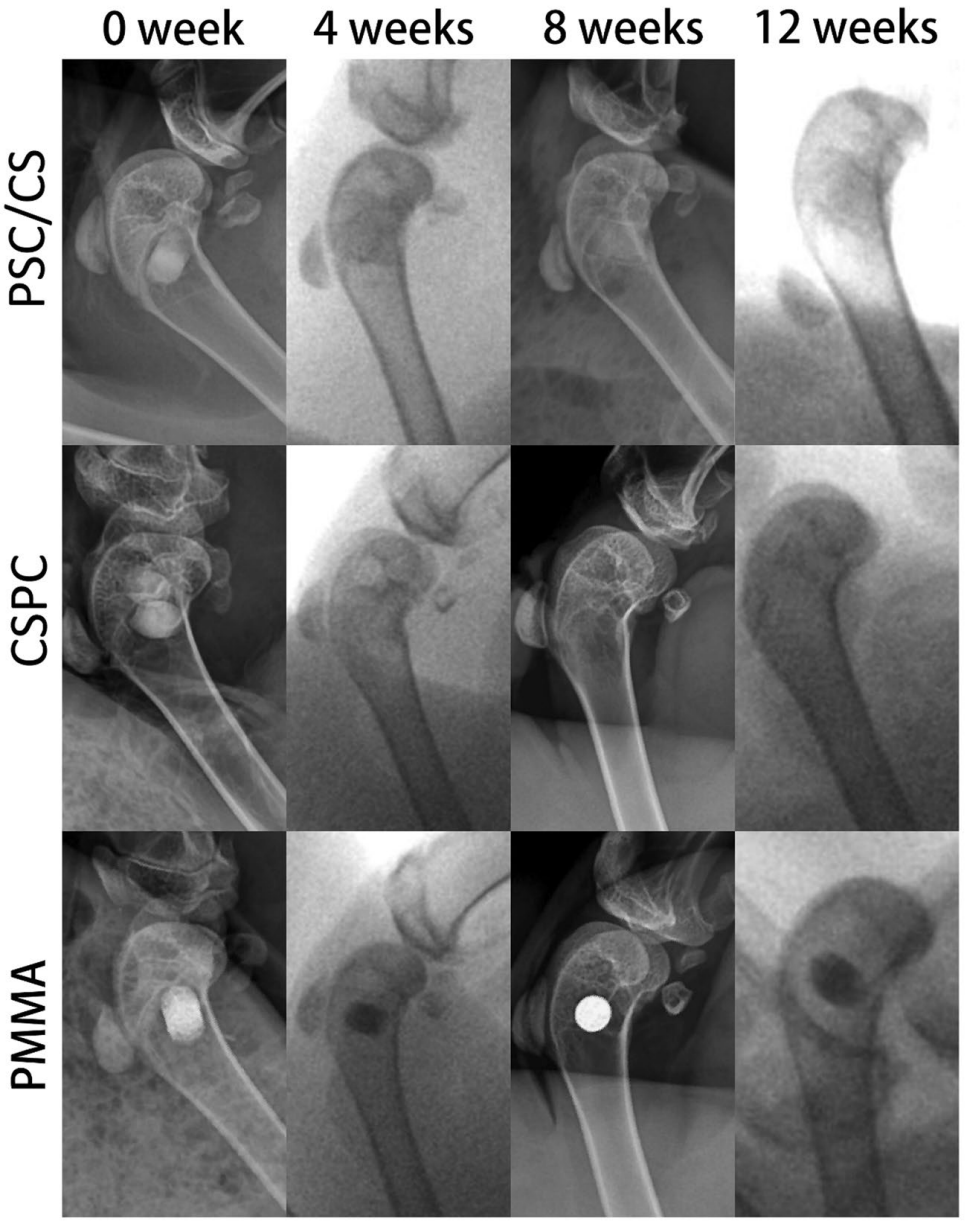
Radiographic images of defect site of three groups.

In Group A with PSC/CS, no significant change observed in the first 4 weeks after the operation. At the 8th week after the operation, the margin of the cement became unclear, indicating the resorption of the cement. At the 12th week, the PSC/CS cement further degraded, and there were some high density signals appeared at the edge of the implanted cement, indicating new bone was formed at the cement-bone interface. In the Group B with CSPC, most of the implanted cement had degraded within the first 4 weeks after the operation. At the 8th week, the implanted cement almost completely degraded and was replaced by new bone. No residual bone cement was observed at the 12th week and the bone defects were partially filled with the newly formed bone. However, in the Group C with PMMA, there was no visible change observed up to the 12th week after the operation (Fig. 3).

#### Micro-computed tomography analysis

**Figure 4:**
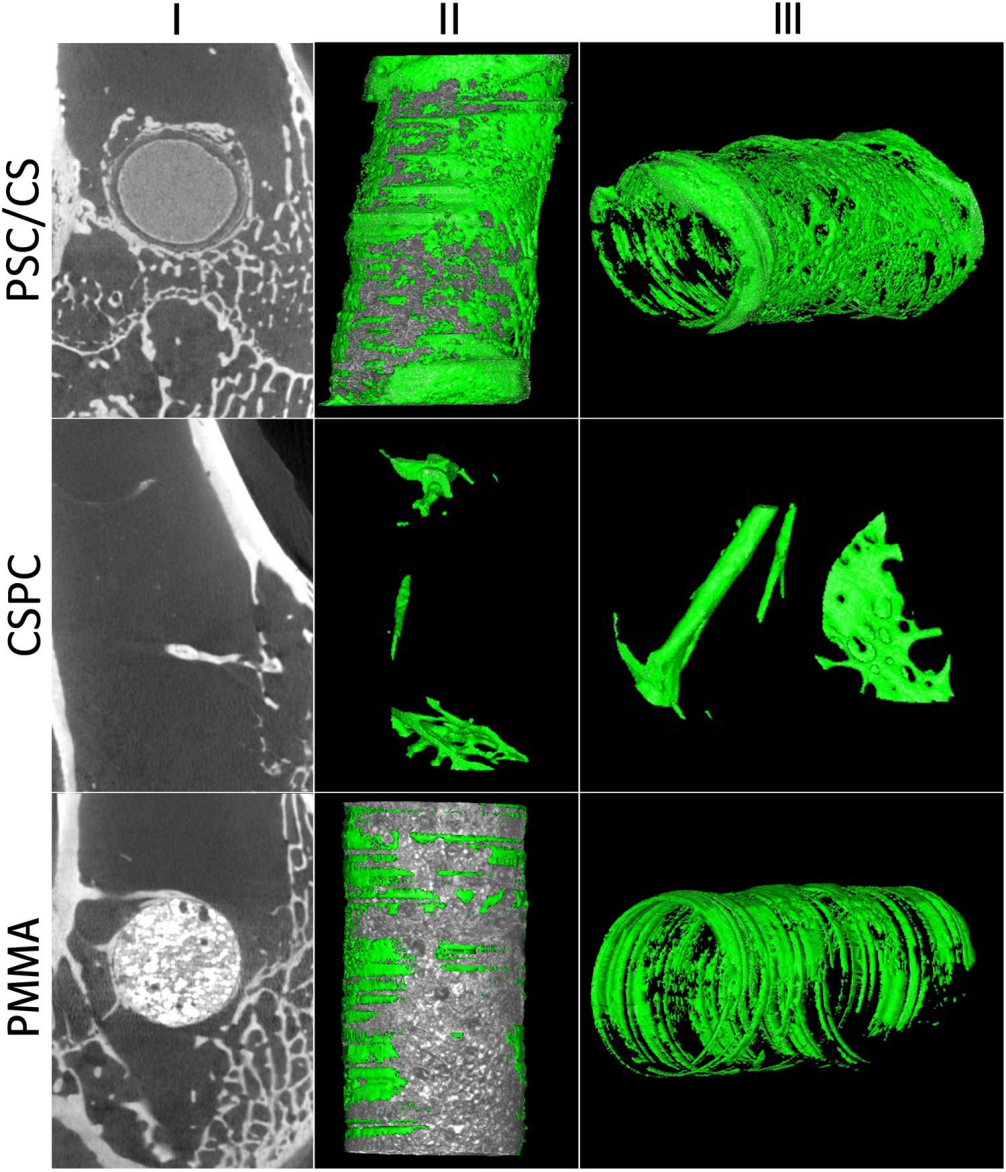
Sagittal images (column I) and 3D reconstructed images (column II and III) by micro-CT imaging of the area surrounding the cement implants after 12 weeks. The green part represents newly formed bone, and the gray part represents residual cement.

Micro-CT images and the 3D reconstruction images of the residual cement as well as the newly formed bone tissue at the defect area were used for evaluation of the *in vivo* osteogenic capacity and the resorption of the cement. In Group A, the PSC/CS degraded partially and the residual material was surrounded by the newly formed bone tissue. In Group B with CSPC, the implanted bone cement almost degraded completely and was partially replaced by the new trabecular. However, in the Group C with PMMA, the bone cement showed few observable changes and only a few newly formed bones appeared at the bone-cement interface (Fig. 4).

**Figure 5:**
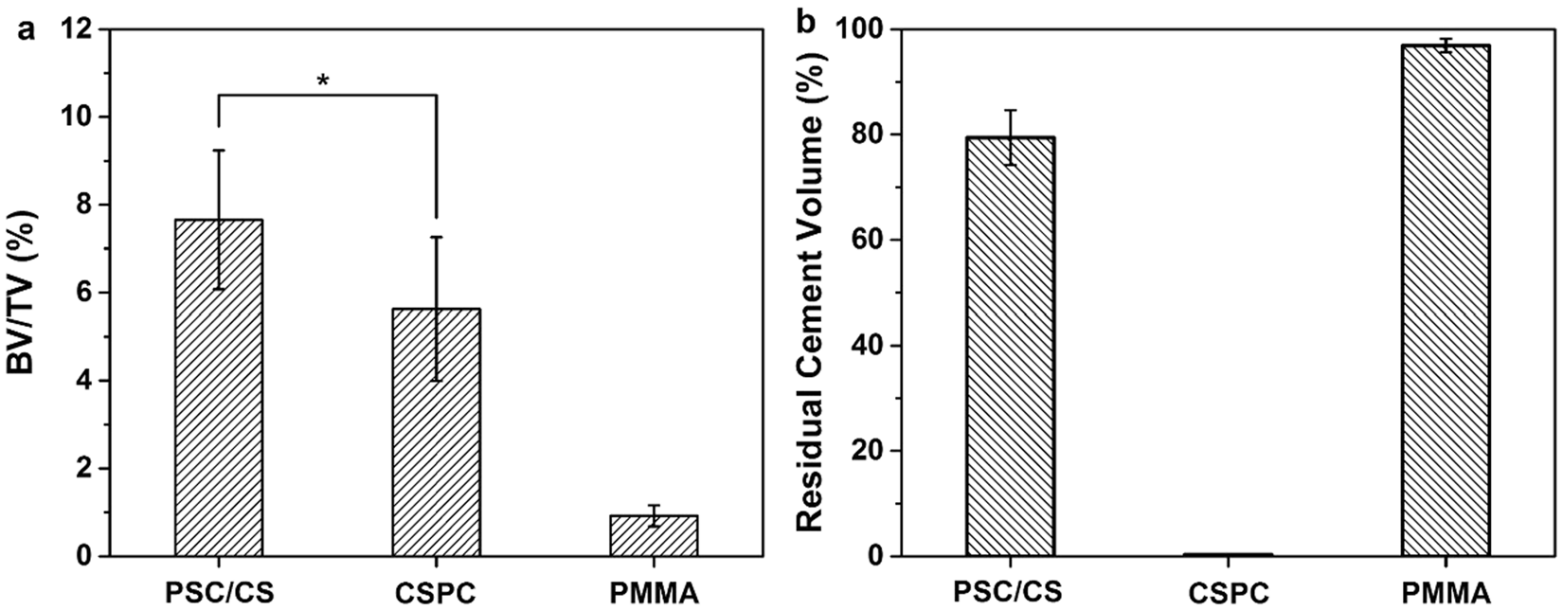
Quantitative analysis of new bone formation and residual cement from micro-CT images. *Indicates significant differences between the groups connected with the line (P < 0.05).

The volume of the new bone and the residual cement within the defect area were calculated to precisely evaluate the bone growth and the resorption rate. As shown in the Fig. 5, at the 12th week, the BV/TV of the PSC/CS (7.7 ± 1.6%) is significantly higher than that of PMMA cement (0.9 ± 0.2%) and CSPC (5.6 ± 1.6%). In addition, the bone cement volume of PSC/CS (79.4 ± 5.2%) was significantly lower than that of the PMMA (96.9 ± 1.3%) at the 12th week after the implantation, and in the Group CSPC, there is no residual bone cement.

The results indicated that the PSC/CS had better capacity to stimulate bone regeneration compared with the CSPC cement and the PMMA cement. The volume of the residual bone cement suggested that compared with the PMMA, PSC/CS had certain ability of degradation. While the resorption rate of the PSC/CS was much lower than that of the CSPC cement. These characters empowered the PSC/CS to generate osteogenesis at the same time of providing biomechanical support in a long-term.

#### Histological evaluation

**Figure 6:**
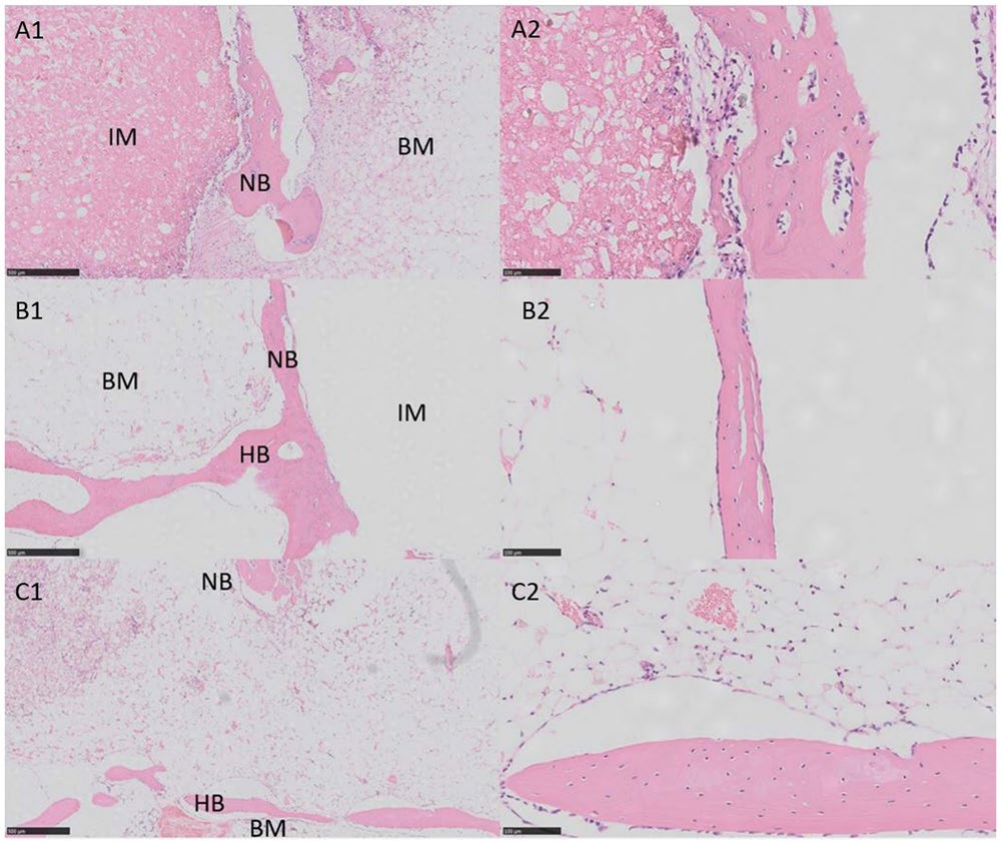
Histology photomicrographs of H&E staining of bone defects for three groups after 12 weeks. Abbreviations and signs used: newly bone (NB), host bone (HB), the implanted material (IM), bone marrow (BM). Scale bar represents 500 μm (left row) and 100 μm (right row).

Histological analysis was also performed to get a more detailed analysis on the new bone formation. Figure 6 showed the photographs of H&E stained sections at the 12th week after surgery. In group PSC/CS, the material partially degraded and the residual materials were surrounded by areas of newly formed bone tissue (Fig. 6a). Compared with the other two groups, there were more osteocyte, trabeculae and vessels at the Bone-Cement interface. In addition, more cells gathered at the interface between the implant materials and the newly formed bone tissue, indicating further osteogenesis comes along with the degrading of the implanted materials. In group CSPC, the implant materials almost degraded completely (Fig. 6b). However, the spaces created by degradation of the materials were not fully replaced with bone tissue. In contrast, only a small amount of bone tissue was observed within the defects. In group PMMA, a thin layer of osteoid formed at the edge of the bone defect site (Fig. 6c). New trabeculae formed at the Bone-Cement interface but there was no bone tissue observed within the defects because of the occupied effect caused by the non-degradability of PMMA.

The result of H&E staining was consistent with the observation by the Micro-CT. It again was confirmed that PSC/CS had a better capacity to stimulate bone regeneration and had certain ability of degradation.

## Discussions

PVP and PKP are now widely used for treating VCFs. For long-term sake, the injectable cements used in PVP and PKP should be biodegradable, thus enabling a total replace of implant by new bone tissue. The currently available degradable bone cements are blamed for the mismatch of bone regeneration rate and implants degradation rate, i.e. the implants disappear before new bone tissue fills the void, thus, insufficient mechanical support is expected. In this study, a new type of BG (PSC) was blended CS to form an injectable cement in order to address the above issue. As mentioned earlier, this new formulation is expected to degradation slower and stimulate bone regeneration better, thus sufficient mechanical support could be achieved during the healing process. All the related properties were evaluated under the context of using this new PSC/CS cement for potential application in PVP or PKP.

In clinic use, setting time, injectability and disintegration resistance are key characteristics for bone cements^22, 23^. According to the results showed in section 3.1, the PSC/CS cement showed excellent injectabiliy (>90%) and stayed homogeneous throughout the whole injection process. Its initial and final setting times were about 25 min and 31 min, respectively, which are longer than the other cements (5–15 mins)^3^. Although a relatively long setting time could offer the surgeons more time for the operation, it is risky because the paste may be washed out by the physiological liquids within this period^2, 24^. Fortunately, this PSC/CS cement showed good resistance to disintegration (D ~ 94%). Even immersed in PBS for 24 h, it can almost maintained the initial shape and no obvious disintegration was observed. These properties indicated that the PSC/CS cement was still a promising candidate for use in minimally invasive surgery.

Ideally, once implanted, the cement should be both bioactive and bioresorbable as well as provide required mechanical support to share the load with the surrounding bone tissues. The compressive strengths (S_c_) and Young’s modulus (E_c_) of the hardened PSC/CS cement were around 2.9 MPa and 340 MPa, respectively, which fulfilled the requirements for cancellous bone substitutes^18, 19^. Fig. 1b showed the *in vitro* degradation behaviors of CSC, CSPC and PSC/CS cements, which clearly highlighted the privilege of PSC/CS cement. The degradation behaviors of the PSC/CS cement seemed to meet the requirements for bone tissue regeneration and mechanical support during healing process.

It has been generally accepted that the formation of HA layer on a bioactive material is essential for the bonding between the material and surrounding tissues^25^. CSC has been criticized for the fact that no HA was deposited on its surface after immersion in SBF due to its non-bioactivity^6, 9, 16^. In comparison, when PSC/CS cement was incubated in SBF, both SEM observation and FTIR investigation revealed the rapid formation of HA on surface, indicating their excellent bioactivity. Furthermore, with the extension of immersion time, the intensity of HA became stronger (Fig. 2a), suggesting the further growth of HA. It should be noted that the deposited HA on surface not only played an important role on bioactivity but also slowed down the degradation rate compared with the pure calcium sulfate.

The implantation of the PSC/CS cement in the critical-sized rabbit femoral condyle defects helps to evaluate its potential in the eventual clinical application. Presently, the most commonly used filling material in PKP and PVP is still the nondegradable PMMA. Because of the lack of osteoinductivity and bioactivity, the long-term stability of the interface between the cement and the bone is not guaranteed^26, 27^. In terms of biocompatibility and bone conductivity, bioresorbable cements such as CSC and CPC are better than PMMA. However, their rapid resorption often results in insufficient persistence of an osteocondutive scaffold to encourage bone apposition as well as the destabilization of early bony apposition^28^, and eventually leads to the recollapse of the fractured vertebra^10^. In the treatment of VCFs, spinal deformity reduction is as important as pain relief^29^, hence requesting the implanted cement to have the ability of osteogenesis while having a reabsorbed rate matched to the rate of new bone formation in order to provide a long-term maintenance of vertebral height restoration. Based on our *in vivo* test, PMMA did not degrade at all and new bone tissue was only found at the bone-cement interface (Fig. 6c). It can stabilize the fracture fragment, thus leading to a rapid pain relief, however, the bone-cement may be loosen in long term due to the lack of bony bonding. CSPC showed a certain ability of osteogenesis, however, because of the rapid resorption, the newly formed bone tissue cannot fully fill the spaces created by degradation of the CSPC (Fig. 6b), which will result in recollapse and fail to maintain the vertebral height restoration. This new PSC/CS cement can significantly enhance the osteogenesis at the interfacial areas and stimulate more bone formation, superior to both PMMA and CSPC cements (Fig. 6a). Besides, PSC/CS resorbed slower than CSPC, thus can provide sufficient mechanical support in the healing process.

The appropriate mechanical properties, better osteoinductivity and suitable resorption rate would have a positive effect on the long-term maintenance of the vertebral height while concurrently promote the fracture healing and reduce the postoperative complications such as new fracture of adjacent vertebral body and recollapse of the fracture vertebral body. Based on the results of the properties determined *in vitro* as well as the femoral condyle defect model in a rabbit, this new PSC/CS cement is a potential substitute of commercial bone cements for application in PVP and PKP. In the future, further investigations will focus on larger animal models.

## Materials and Methods

### Materials

PSC powers with the composition of 10.8%P_2_O_5_-54.2%SiO_2_-35%CaO (mol. %) were prepared by a sol–gel method according to our previous work^30^. The obtained powders were ground to form particles of size <38 μm and stored in a desiccator until usage. α-Calcium sulfate hemihydrate (CSH, ≥97.0%) and Tricalcium phosphate (TCP) were purchased from Sigma-Aldrich (St. Louis, MO, USA) and used without further purification. PMMA cement was purchased from Medtronic Inc.

### Preparation of PSC/CS cements

The PSC/calcium sulfate composite cements (PSC/CS cements) were prepared by combining a solid and a liquid phase. The solid phase was prepared by mixing the obtained PSC and CSH powers with PSC content of 55 wt %. The setting-liquid phase was a chitosan solution that prepared according to the literature^31^. Then, the solid powders were mixed with setting-liquid phases for 1 min with a spatula at the L/P ratio of 0.5 ml/g to get a homogeneous cement pastes.

CSC and calcium sulfate/calcium phosphate composite cement (CSPC, CSH: TCP = 1:1) samples were also prepared as a comparison following the same procedure for PSC/CSC cements.

### Characterization of PSC/CS cement

#### Injectability

The injectability of composite cements was tested according to the literatures^32, 33^. Briefly, approximately 4 g of the homogeneous cement pastes prepared as described above were put into a 5 ml syringe with an opening nozzle diameter of 2 mm. After 3 min since the beginning of mixing the composite powders and liquid, the cement was extruded by hand until it was too hard to push the syringe. The percentage of cement that could be extruded from the syringe is used to evaluate the injectability coefficient (J), as (1):

1 $$J( \% )=\frac{{M}_{1}-{M}_{2}}{{M}_{1}-{M}_{0}}\times 100 \% $$

where *M*_*0*_ is the mass of the empty syringe, *M*_*1*_ is the total mass of syringe and cements, and *M*_*2*_ is the remaining mass of syringe and cements after extrusion.

#### The disintegration resistance

The degree of cohesion or the disintegration resistance of cements in liquid was tested by injecting 1 g newly prepared cement into phosphate-buffered saline (PBS, pH = 7.2 ~ 7.4) at 37 °C. After soaking in PBS for 24 h, the integrity of cement was observed by naked eyes and the amount of non-decayed cement was carefully collected, dried and weighed (*W*_*1*_). Another 1 g newly prepared cement were also dried and weighed (*W*_*2*_) as a control. Each measurement was performed in triplicate. The disintegration resistance (D) was determined using the equation (2):

2 $$D( \% )={W}_{1}/{W}_{2}\times 100 \% $$

#### The setting time and Mechanical Properties

The setting time of the composite cements were measured with a Vicat apparatus, according to ISO 9597:2008(E). The mechanical properties of PSC/CS cements (Ø4.5 × 9.0 mm) were performed on a universal testing machine (Instron3365, Instron Co., Canton, MA, USA) with a 5kN load cell at a loading rate of 0.5 mm/min until sample failure. At least 5 samples of each cement were tested, and the results were expressed as a mean ± SD.

### *In Vitro* Bioactivity and degradability

The *in vitro* bioactivity and degradability of cements were evaluated by soaking in simulated body fluid (SBF)^33^. The hardened cylinder samples (Ø 4.5 × 4.0 mm) were immersed in SBF with a surface area-to-volume ratio of 0.1 cm^−1^ and kept at 37.0 °C. The surface morphology and chemical structures of the PSC/CS before and after SBF immersion were measured using scanning electron microscopy (SEM, JEOL-6700) and Fourier transform infrared (FTIR, Bruker Equinox 55 instrument) to examining the ability of apatite formation on their surfaces.

The initial weight (*W*_*0*_) of each sample was recorded. After soaking in the SBF for different periods, the specimen were taken out, washed and dried at 60 °C until constant weight. The final weight (*W*_*t*_) of the dried specimen was monitored. The degradation was determined by weight loss according to the following equation (3):

3 $$Mass\,Loss( \% )=\frac{{W}_{0}-{W}_{t}}{{W}_{0}}\times 100 \% $$

### Cell Toxicity and Attachment

MG63 cells were used to investigate the biocompatibility of cements.

#### 4.5.1 Cell toxicity

The specimen of each cement were previously sterilized by autoclaving at 180 °C for 2 h and then soaked in α-MEM, preheated to 37 °C (extracting vehicle ratio = 12.5 mg/mL), for 3 days. Under sterile conditions, α-MEM was filtered to eliminate solid cements and these extracts were used as culture medium after adding 10 vol. % fetal bovine serum (FBS) and 1 vol. % penicillin/streptomycin antibiotics. MG63 cells with a density of 2000 cells/plate were seeded onto 96-well culture plates and incubated in the cell incubator. After cell adhesion was verified, the culture medium was replaced by the extracts of different cements. The cells incubated in a-MEM without extract were used as a control. After culturing for 48 h, cell viability on the samples was assessed quantitatively by the thiazolyl blue (MTT) assay. The optical density (O.D. value) of each sample was measured at 490 nm. Five specimens in each group were tested.

#### Cell attachment

In a parallel experiment, the samples (Ø 12 × 2 mm) were sterilized by autoclaving at 180 °C for 2 h and then put in each well of the 24-well plate. MG63 cells with a density of 5 × 10^4^ cells/sample were seeded onto the samples, and incubated in the cell incubator. At 24 h, the cell-cements were gently rinsed with PBS three times, and fixed with 2.5% glutaraldehyde at 4 °C for 12 h. The cell-cements were sequentially dehydrated in graded ethanol solutions (50, 75, 95, and 100 wt %), dried and the cell attachment and morphology were directly observed by SEM.

### *In vivo* evaluation of cement in a rabbit femoral condyle defect model

#### Surgical procedure

All the animal experimental procedures were approved by the Animal Research Committee of Peking University (Permit number: LA201415) and we conformed that all methods were performed in accordance with the relevant guidelines and regulations. In total there were 12 adolescent male New Zealand white rabbits weighing 3.0–3.5 kg used and all of them were anesthetized using an ketamine hydrochloride (50 mg/kg, IM) and fentanile (0.17 mg/kg, IM). The distal femoral epiphysis exposure was performed using lateral approach, and a critical-sized condyle defect (5 mm in diameter and 10 mm in depth)^34, 35^ was created using an electric trephine (Johnson & Johnson, USA) under irrigation with 0.9% sterile saline solution. The defect model was treated using three kinds of injectable bone cement: the novel PSC/CS and two commercially available bone cements: PMMA cement (Medtronic, America) and CSPC cement (CSH: TCP = 1:1). The left femoral condyle defect of all the rabbits were implanted with the PSC/CS (Group A), while the right femoral condyle defect of six rabbits were treated with the CSPC (Group B), and the right femoral condyle defect of the other six were treated with the PMMA (Group C). The wounds were sutured well and the prophylactic antibiotic together with the analgesics was given for three days.

#### Radiographic examination

After the surgery, rabbits were fastened in the lateral position under anesthesia for the X-ray radiographs of the bone defect after 0, 4, 8 and 12 weeks. In order to precisely evaluate the defect healing process, the radiographs of each rabbit were inspected by at least two experienced orthopedic surgeons who did not participate in the study and were blinded from the detailed experimental process.

#### Micro CT evaluation

For microcomputed tomography evaluation, all the rabbits were sacrificed by overdose of pentobarbital at the 12th weeks after surgery. The rabbit femurs were examined by the Micro-CT (Inveon Scanners, SIEMENS, German) after removing the soft tissue attached to the femur. To evaluate the *in vivo* resorption of the implanted bone cement, the residual bone cement volume fraction was calculated as the ratio between the volume of residual cement and the defined VOI (volume of interest). And the amount of newly formed bone was quantified as the bone volume (BV) within the defined VOI (volume of interest) in each defect site by using a CT analyzing software (Inveon Research Workplace, SIEMENS, German).

#### Histologic analysis

After the radiographic examination and Micro CT evaluation, the harvested femur were washed with saline thoroughly, then fixed in 4% paraformaldehyde (10% neutral buffered formalin) for no more than 72 h and subsequently decalcified for up to 6 weeks in 10% EDTA, pH 7.0 at 4 °C. After complete decalcification and dehydration, the samples were embedded in paraffin wax. Then 5 mm serial slices were prepared using a microtome, subsequent surface staining was performed with haematoxylin and eosin (H&E) for microscopic observation. The slides were photographed using a digital camera (NanoZoomer-SQ, Hamamatsu Photonics K.K., Hamamatsu, Japan).

### Statistical analysis

Statistical analysis was performed using one-way ANOVA and the Student’s t-test. All quantitative data were presented as mean ± SD. Differences were considered statistical significant at p < 0.05.

### Data Availability

All data submitted in the manuscript are available.

## Conclusion

In this study, a new PSC/CS composite cement with high content of PSC was developed and evaluated both *in vitro* and *in vivo*. Excellent injectability, good resistance to disintegration and radiopacity were observed from this composite cement, which all made it easier for operation. The *in vitro* test showed that the cement was bioactive and had suitable mechanical properties. More importantly, under physiological conditions, it could maintain its shape sustainably and possibly provide a long-term mechanical support for bone regeneration. In addition, this cement showed a better capacity than the PMMA and CSPC in terms of bone regeneration as well as the resorption rate observed in a critical-sized rabbit femoral condyle defect model. Based on these observations, this cement is a promising bone cement to treat the VCFs using minimally invasive surgery.

## Electronic supplementary material

csc video

cspc video

psccs video

Supporting information
