# Supplementary material for: Novel bioactive glass based injectable bone cement with improved osteoinductivity and its in vivo evaluation
Source: Sci Rep. 2017 Jun 15;7:3622. doi: 10.1038/s41598-017-03207-9 (PMC5472605; doi:10.1038/s41598-017-03207-9)
Supplement: Supplementary file 4 — Supporting information [file 41598_2017_3207_MOESM4_ESM.docx]

**Novel bioactive glass based injectable bone cement with improved** **osteoinductivity and its in vivo evaluation**

Tengjiao zhu , Huihui Ren, Ailing Li, Bingchuan Liu, Caiyun cui, Yanmei Dong, Yun Tian, Dong Qiu

Supporting information


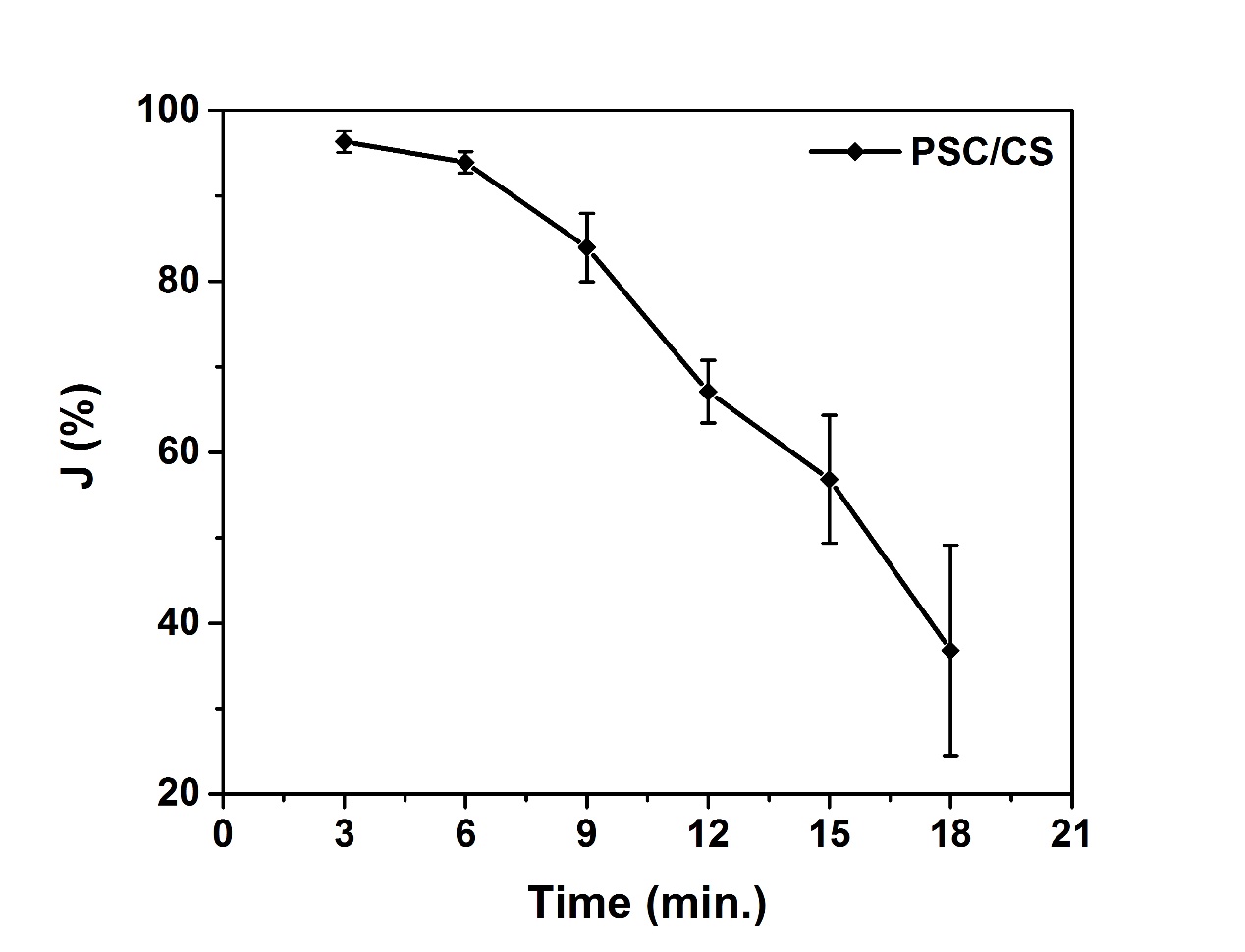


Fig. S1. The injectability of PSC/CS cement as a function of time.

Fig. S2. The behaviors of CSC, CSPC, and PSC/CS cement injected into PBS.

（Video CSC,CSPC,PSCCS-cement）
